# Laparoscopic Surgery in Elderly Patients Aged 65 Years and Older with Gynecologic Disease

**DOI:** 10.5402/2012/678201

**Published:** 2012-11-21

**Authors:** Haruhiko Kanasaki, Aki Oride, Kohji Miyazaki

**Affiliations:** Department of Obstetrics and Gynecology, School of Medicine, Shimane University, 89-1 Enya Cho, Shimane, Izumo 693-8501, Japan

## Abstract

*Objectives*. The study was conducted to characterize the use of the laparoscopic surgery in elderly patients. *Methods*. The medical records of elderly patients aged ≥65 years who underwent laparoscopic surgery were retrospectively reviewed for diseases, surgical procedures, histological diagnosis, intraoperative and postoperative complications, and reasons for presentation. *Results*. Of the 405 patients who underwent laparoscopic surgery between January 2005 and March 2012, 41 (10.1%) were aged ≥65 years. The most common disease treated by surgery was ovarian tumor, followed by uterine prolapse. Histological diagnosis of ovarian tumor specimens obtained from 23 patients included serous cystadenoma (44.0%), mature cystic teratoma (20.0%), mucinous cystadenoma (20.0%), and endometrioma (4%). In contrast, in the non-elderly group, the most common histological diagnosis was endometrioma (42.9%), followed in order by mature cystic teratoma (28.3%), serous cystadenoma (18.0%), and mucinous cystadenoma (4.7%). While 23.7% of the non-elderly patients required emergency laparoscopic surgery, none of the elderly patients required emergency surgery. Only 1 of 27 patients who underwent surgery for an ovarian or adnexal mass presented with abdominal pain. No one developed serious intraoperative or postoperative complications. *Conclusion*. Laparoscopic surgery can be safely performed in elderly patients. It should be noted, however, that few elderly patients with benign pelvic mass manifest symptoms before undergoing surgery.

## 1. Introduction

Laparoscopic surgery is widely performed for the treatment of benign gynecologic diseases as it is associated with lower invasiveness, less pain, and shorter postoperative hospital stay than open surgery [1, 2]. In Japan, where the population is rapidly aging and life expectancy is increasing, laparoscopic surgery is expected to be performed more frequently in elderly women with gynecologic disease.

In the field of gastrointestinal surgery, where many patients are elderly, laparoscopic surgery has been extensively used for elderly patients undergoing surgeries such as cholecystectomy and resection of digestive tumors since the 1990s, with fewer complications reported compared with open surgery [3, 4]. In the field of gynecology, the use of laparoscopy was initially intended for intraperitoneal examination of infertile women and was later extended to surgical treatment of conditions commonly affecting younger women, such as benign ovarian cyst and ectopic pregnancy. The selection of laparoscopic versus open surgery therefore takes into account esthetic considerations, and there have been a limited number of studies on the use of laparoscopic surgery in elderly patients with gynecologic disease.

This study aimed to characterize and evaluate the safety of laparoscopic surgery in elderly patients with gynecologic disease by retrospectively analyzing the medical records of elderly female patients aged ≥65 years who underwent laparoscopic surgery during a 7-year period between January 2005 and March 2012.

## 2. Methods

A retrospective medical record review was conducted at Shimane University Hospital in Shimane prefecture, Japan. The medical records of all women who underwent laparoscopy for gynecologic disorders at some time between January 1, 2005 and March 31, 2012 (*n* = 405) were reviewed. Medical history was taken by a physician, and all patients gave informed consent for the surgery. A single investigator reviewed each record and abstracted the data.

 While no upper age limit was defined for selecting candidates for laparoscopic surgery, elderly patients with impaired function of major organs, were bedridden, or had advanced dementia were excluded. The age of ≥65 years is generally considered as the stage of old age and therefore, for the purpose of the present study, we identified elderly patients aged ≥65 years who underwent laparoscopic surgery in the past 7 years. Their medical records were analyzed for diseases treated by surgery, surgical procedures, histological diagnosis of resected specimens, events that led to surgery, and intraoperative and postoperative complications. 

In laparoscopy surgery, after general anesthesia, a 10 mm trocar was inserted just below the umbilicus and insufflated with carbon dioxide at a pressure of 8–10 mm Hg. On direct view, two trocars (12 or 5 mm) were placed through lower abdominal incisions. In some cases, single-port surgery was applied. 

## 3. Results

Of the 405 patients who underwent laparoscopic surgery between January 2005 and March 2012, 41 (10.1%) were aged ≥65 years. One patient, a 66-year-old woman with benign ovarian cyst in whom laparoscopic procedure was converted to open surgery after severe intraperitoneal adhesion was identified during the procedure, was excluded from the analysis.

 The diseases treated by surgery and surgical procedures used in the 41 elderly patients are summarized in Table 1. The most common disease treated by surgery was ovarian tumor in 21 (51.2%) patients, followed by uterine prolapse in 8 (19.5%) patients and uterine fibroids in 5 (12.2%) patients. Two patients had ovarian tumor and uterine prolapse and 1 patient had paraovarian cyst and uterine prolapse. Surgical procedures included bilateral salpingo-oophorectomy (BSO) in 17 (41.5%) patients, laparoscopically assisted vaginal hysterectomy (LAVH) combined with BSO in 12 (29.3%) patients, unilateral salpingo-oophorectomy in 7 (17.1%) patients, and enucleation of uterine fibroids in 4 (9.8%) patients. One patient who developed pyometra due to cervical cancer-related cervical obstruction underwent laparoscopic uterine drainage (puncture and pus drainage).

 Among the 41 elderly patients who underwent laparoscopic surgery, ovarian tumor was the most common disease treated by the procedure, performed in 23 patients, including 2 patients who had concomitant uterine ptosis. The histological diagnoses of 25 ovarian tumor specimens obtained from the 23 patients, including those from 2 patients with bilateral ovarian tumors, are summarized in Table 2. The most common diagnosis was serous cystadenoma in 11 (44.0%) patients, followed in order by mature cystic teratoma in 5 (20.0%), mucinous cystadenoma in 5 (20.0%), simple cyst in 2 (8.0%), and endometrioma in 1 (4.0%) patient. Among 233 non-elderly patients aged <65 years who underwent laparoscopic surgery for ovarian tumor during the same period, the most common histological diagnosis was endometrioma in 100 (42.9%) patients, followed in order by mature cystic teratoma in 66 (28.3%) patients, serous cystadenoma in 42 (18.0%) patients, and mucinous cystadenoma in 11 (4.7%) patients.

 In the elderly group, none of the 41 patient required emergency laparoscopic surgery (defined as being performed within 12 h after presentation). In contrast, in the non-elderly group, 23.7% of the 363 patients required emergency surgery.

 Background data were then analyzed for the 27 elderly patients, including 23 patients with ovarian tumor and 4 patients with paraovarian/tubal cyst, who were at risk of developing ovarian torsion. The preoperative tumor diameter range of 5–9 cm accounted for the largest portion, 14 (51.9%) patients, while 6 (22.2%) patients had tumors of ≥10 cm and 7 (25.9%) patients had tumors of ≤4 cm (Table 3). The reason for seeing a gynecologist was incidental discovery of a tumor on ultrasonography during gynecologic cancer screening for 12 (44.4%) patients, incidental discovery on examination by another department for 10 (37.0%) patients, and discovery of a tumor following presentation to their gynecologists with irregular genital bleeding or other gynecologic disorder for 4 (14.8%) patients. Only 1 (3.7%) patient presented due to abdominal pain (Table 4).

 Of the 5 patients who underwent laparoscopic surgery for uterine fibroids, 4 underwent enucleation of fibroids and 1 underwent LAVH. In 4 of the 5 patients, surgery was performed due to suspected ovarian tumor. The remaining patient was diagnosed with suspected uterine fibroids by positron emission tomography and LAVH was performed respecting the patient's wishes, although subsequent histological examination revealed that the lesion was benign leiomyoma. None of the 5 patients who underwent surgery for uterine fibroids complained of abdominal pain prior to presentation. These patients were referred to our hospital after fibroids were incidentally discovered during gynecologic cancer screening or examination at another department (Table 4).

 None of the 41 elderly patients who underwent laparoscopic surgery developed significant intraoperative or postoperative complications. One patient developed extrasystole during surgery and was treated pharmacologically and another patient developed hypertension during surgery and was also treated pharmacologically. In addition, 1 patient with systemic lupus erythematosus postoperatively developed norovirus infection and impaired kidney function. 

## 4. Discussion

In an increasingly aging society, laparoscopic surgery is increasingly being needed for elderly patients in the field of gynecology. However, only a limited number of studies have been conducted on laparoscopic surgery in elderly patients with gynecologic disease. Compared to younger patients, elderly patients are more likely to have existing conditions when undergoing surgery, as well as reduced major organ function and reduced functional reserve for invasive surgery [5, 6]. The use of laparoscopic surgery for elderly patients appears to be prudent due to possible hemodynamic and respiratory effects caused by pneumoperitoneum. The definition of the elderly remains somewhat controversial; in Japan, those aged ≥65 years are generally considered as being elderly and thus we examined the data for this population here. 

 The most common diseases treated by surgery were benign ovarian tumors, which accounted for more than half of the cases and were treated by salpingo-oophorectomy, followed in order by uterine prolapse and uterine fibroids, which were treated by LAVH and myomectomy, respectively. The use of laparoscopic surgery for ovarian tumors and uterine fibroids is common in the gynecological field and is thus not specific to the elderly population, whereas the use of LAVH for uterine prolapse is specific to patients in this age group. In cases of ovarian tumor, we did not perform tumor enucleation, an ovary-preserving procedure, and instead performed unilateral or BSO in all cases. This decision was made based on the same general criteria as used in cases of open surgery.

 The most common histological diagnosis of ovarian tissue specimens obtained from the 23 patients with ovarian tumor was serous cystadenoma, accounting for 44%, while only 1 patient was diagnosed as having endometrioma. In contrast, in the group of patients aged <65 years, 42% of the patients underwent laparoscopic surgery for endometrioma and 18% underwent it for serum cystadenoma. This may reflect the differential histological diagnosis of ovarian tumors between age groups. Interestingly, none of the elderly patients with pelvic tumor required emergency surgery due to acute abdomen caused by such conditions as ovarian torsion/rupture. In the non-elderly group, 40 of the 233 (17.2%) patients who underwent laparoscopic surgery for ovarian tumor required emergency surgery due to abdominal pain. This is also clearly reflected in the reasons for presentation to a gynecologist that led to surgery. All patients with pelvic mass were incidentally found to have gynecologic disease during gynecologic screening or examination at another department, except for 1 patient who presented with abdominal pain. This patient also had only mild pain that did not require an emergency surgical procedure and she underwent elective surgery more than 2 weeks after presentation. We have previously reported that 20% of patients with mature cystic teratoma and 5% of those with serous cystadenoma required emergency laparoscopic surgery [7]. Given that more than half of the patients had tumors >5 cm, it appears that elderly patients with pelvic mass are less likely to develop symptoms accompanying abdominal pain, regardless of mass size. Of the 5 patients who underwent laparoscopic surgery for uterine fibroids, 4 underwent surgery for suspected ovarian tumor, although none of them had subjective symptoms such as pain, and all of them were incidentally found to have a mass during gynecologic screening or examination at another department. Thus, when a mass has been incidentally found in an elderly patient with no subjective symptoms, it may be an option to simply follow the patient without surgical intervention, provided that the possibility of malignancy can be ruled out. 

 The use of laparoscopic surgery for elderly patients was first applied in the area of gastrointestinal surgery for cholecystectomy, and many of the previous studies on this procedure have shown no significant difference in the incidence of complications or postoperative hospital stay between elderly and younger patients and a lower incidence of complications with laparoscopic surgery than with open surgery [3, 4]. Laparoscopic surgery for benign gynecologic disease has also been shown to be safe whether the patient is elderly or young and has been shown to be beneficial as it is associated with lower surgical invasiveness and less postoperative pain compared to open surgery in elderly patients [8, 9]. We also observed no surgical complications during surgery in the present series, except for 1 case of intraoperative arrhythmia and extrasystole and another case of intraoperative hypertension. The only patient who developed postoperative complications had systemic lupus erythematosus and was on steroid therapy prior to surgery. In this case, surgical stress caused further impairment of kidney function. 

 In conclusion, laparoscopic surgery can be safely performed in elderly patients with benign gynecologic disease and is associated with a low incidence of respiratory and cardiovascular complications, major concerns associated with laparoscopic surgery in elderly patients, as also supported by previous reports. For the treatment of benign disease in elderly patients, laparoscopic surgery is recommended due to its lower surgical invasiveness and lower intensity of postoperative pain compared to open surgery. However, since pelvic masses such as ovarian tumor and ovarian fibroids rarely cause abdominal pain in elderly patients, surgical indication should be determined carefully respecting the patient's wishes.

## Figures and Tables

**Table 1:** Diseases treated by surgery and surgical procedures in 41 elderly patients.

	No.	%
Diseased treated by surgery		
Ovarian tumor	21	51.2
Uterine prolapse	8	19.5
Uterine leiomyoma	5	12.2
Ovarian tumor + uterine prolapse	2	4.9
Paraovarian cyst	2	4.9
Paraovarian cyst + uterine prolapse	1	2.49
Paratubal cyst	1	2.49
Pyometra (cervical cancer)	1	2.49

Total	41	100

Surgical procedure		
BSO	17	41.5
LAVH + BSO	12	29.3
USO	7	17.1
Myomectomy	4	9.8
Uterine drainage	1	2.4

Total	41	100

**Table 2:** Histological diagnosis of ovarian tumor.

Histology of ovarian tumor	Age ≥65 years	%	Age <65 years, *n*	%
Serous cystadenoma	11	44.0	42	18.0
Mature cystic teratoma	5	20.0	66	28.3
Mucinous cystadenoma	5	20.0	11	4.7
Simple cyst	2	8.0	5	2.2
Endometrioma	1	4.0	100	42.9
Other	1	4.0	9	3.9

Total	25	100	233	100

**Table 3:** Diameter of ovarian tumor or paraovarian/tubal cyst.

Diameter	Number	%
≤4 cm	7	25.9
5–9 cm	14	51.9
10–14 cm	5	18.5
≥15 cm	1	3.7

Total	27	100

**Table 4:** Clinical background of elderly patients.

	Adnexal tumor		Uterine leiomyoma	
	Number	%	Number	%
Incidentally found during screening	12	44.4	3	60
Presented to gynecologist with other symptom	4	14.8	0	0
Found during examination at another department	10	37.0	2	40
Presented to gynecologist with abdominal pain	1	3.7	0	0

Total	27	100	5	100
